# Genome-wide association study of hyperthyroidism based on electronic medical record from Taiwan

**DOI:** 10.3389/fmed.2022.830621

**Published:** 2022-07-27

**Authors:** Ting-Yuan Liu, Wen-Ling Liao, Tzu-Yuan Wang, Chia-Jung Chan, Jan-Gowth Chang, Yu-Chia Chen, Hsing-Fang Lu, Hsien-Hui Yang, Shih-Yin Chen, Fuu-Jen Tsai

**Affiliations:** ^1^Center for Precision Medicine, China Medical University Hospital, Taichung, Taiwan; ^2^Million-Person Precision Medicine Initiative, Department of Medical Research, China Medical University Hospital, Taichung, Taiwan; ^3^College of Chinese Medicine, Graduate Institute of Integrated Medicine, China Medical University, Taichung, Taiwan; ^4^Center for Personalized Medicine, China Medical University Hospital, Taichung, Taiwan; ^5^Genetics Center, Medical Research, China Medical University Hospital, Taichung, Taiwan; ^6^Department of Internal Medicine, College of Medicine, China Medical University, Taichung, Taiwan; ^7^Division of Endocrinology, China Medical University Hospital, Taichung, Taiwan; ^8^China Medical University Beigang Campus, Yunlin, Taiwan; ^9^School of Chinese Medicine, China Medical University, Taichung, Taiwan; ^10^Department of Medical Genetics, China Medical University Hospital, Taichung, Taiwan

**Keywords:** genome-wide association study (GWAS), phenome-wide association studies (PheWAS), hyperthyroidism, electronic medical record (EMR), stroke

## Abstract

Excess thyroid hormones have complex metabolic effects, particularly hyperthyroidism, and are associated with various cardiovascular risk factors. Previous candidate gene studies have indicated that genetic variants may contribute to this variable response. Electronic medical record (EMR) biobanks containing clinical and genomic data on large numbers of individuals have great potential to inform the disease comorbidity development. In this study, we combined electronic medical record (EMR) -derived phenotypes and genotype information to conduct a genome-wide analysis of hyperthyroidism in a 35,009-patient cohort in Taiwan. Diagnostic codes were used to identify 2,767 patients with hyperthyroidism. Our genome-wide association study (GWAS) identified 44 novel genomic risk markers in 10 loci on chromosomes 2, 6, and 14 (*P* < 5 × 10–14), including CTLA4, HCP5, HLA-B, POU5F1, CCHCR1, HLA-DRA, HLA-DRB9, TSHR, RPL17P3, and CEP128. We further conducted a comorbidity analysis of our results, and the data revealed a strong correlation between hyperthyroidism patients with thyroid storm and stroke. In this study, we demonstrated application of the PheWAS using large EMR biobanks to inform the comorbidity development in hyperthyroidism patients. Our data suggest significant common genetic risk factors in patients with hyperthyroidism. Additionally, our results show that sex, body mass index (BMI), and thyroid storm are associated with an increased risk of stroke in subjects with hyperthyroidism.

## Introduction

Hyperthyroidism is a common endocrine disorder with a prevalence of ~0.3–0.5% in an iodine-replete area (1, 2). Excessive amounts of thyroid hormones have profound effects on the cardiovascular system (3). Hyperthyroidism can cause increased heart rate, contractility, wide pulse pressure, systolic hypertension, changes in peripheral vascular resistance, and predisposition to dysrhythmias (3, 4).

In Taiwan, the prevalence of hyperthyroidism is ~2% (5). Autoimmune thyroid diseases account for 40–70% of hyperthyroidism sufferers, including Graves' disease and Hashimoto's thyroiditis. The remainder includes hyperfunctioning thyroid adenomas, subacute thyroiditis, thyroid cancer, and pituitary tumors (6, 7).

Although hyperthyroidism may involve both short- and long-term cardiovascular consequences (8), data concerning the association between hyperthyroidism and cardiovascular outcomes are inconsistent (9). Thyroid dysfunction, which leads to effects on the cardiovascular system and increases an individual's risk of death, is currently under debate. In particular, there is few data to demonstrate that hyperthyrodism increase the risk of stroke in young adults.

It is well-known that genetic factors play an important role in disease etiology and pathogenesis (10). Genetic diseases result from the accumulation of genetic alterations. Therefore, genetic alterations could serve as effective biomarkers for the early detection, monitoring, and prognosis of genetic diseases. In the present study, we summarize the accumulation and achievements of big genomic data and show how they could contribute to precision medicine by using hyperthyrodism as a genetic disease model.

## Methods

### Data sources and informed consent

The China Medical University Hospital Precision Medicine Project was initiated in 2018 and remains operational. This project was approved by the ethical committees of CMUH (CMUH107-REC3-058 and CMUH110-REC3-005). Thus, far, more than 170,000 people have contributed. In this study, all clinical information was collected from the electronic medical records (EMRs) of CMUH and approved by the respective ethical committees of CMUH (CMUH110-REC1-095). The EMR data were collected between 1992 and 2019.

### SNP array data quality control

**Figure 1:**
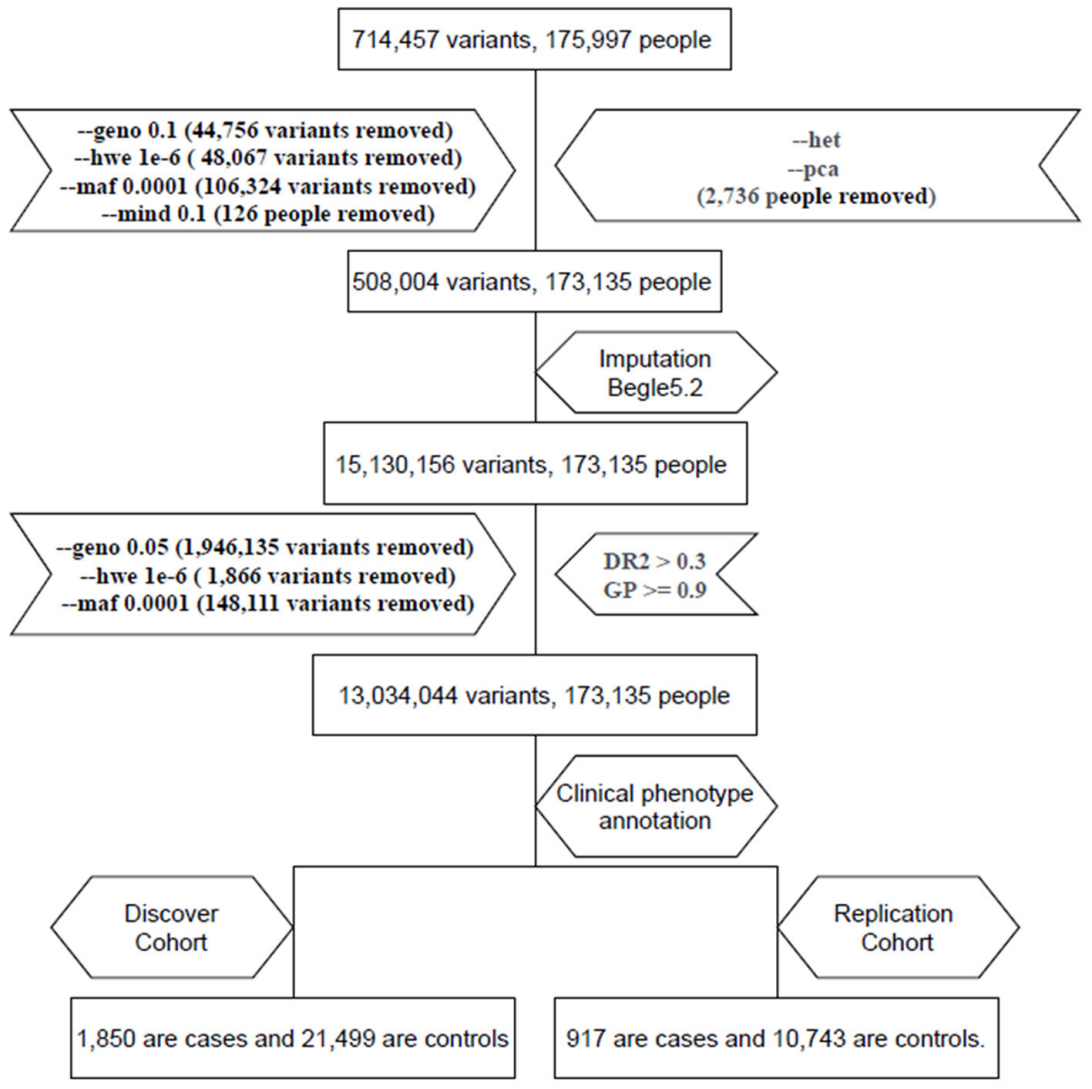
Flow chart of electronic medical record (EMR) data mining and the genome-wide association studies (GWAS) (GWAS) pipeline. We enrolled two cohorts: one with 1,850 hyperthyroidism patients and 21,499 age and sex matched individuals and a replication cohort with 917 hyperthyroidism patients and 10,743 age and sex matched individuals.

We used the TPMv1 customized SNP array (Thermo Fisher Scientific, Inc., Santa Clara, CA, USA), which was designed from the Academia Sinica and Taiwan Precision Medicine Initiative (TPMI) teams. The SNP array contained approximately 714,457 SNPs. PLINK1.9 (11) was used for the analysis. We excluded subjects and SNPs with missing rates (subjects excluded: missingness per marker –geno 0.1 > 10% for SNPs and missingness per individual –mind 0.1 >10% for subjects). We filtered out variants with a Hardy–Weinberg equilibrium *p* < 10^−6^ (–hwe 10^−6^) and minor allele frequency (MAF) of <10^−4^ (–maf 0.0001). Therefore, 508,004 variants and 173,135 subjects passed the filters and the quality control process; then, we used Beagle 5.2 to impute. The imputed data were filtered out using an alternate allele dose of <0.3 and a genotype posterior probability of <0.9 as the criteria (12). After the quality control and imputation process, we analyzed 13,034,044 variants and 173,135 subjects (13) (Figure 1).

### Genome-wide association study

We used PLINK 1.9 for the summary statistics. Subjects who had been diagnosed with hyperthyroidism three times in the EMR were defined as cases. These patients also included those taking medications for hyperthyroidism. Data included values from thyroid-related tests (free T4 and TSH). Subjects who had never been diagnosed with thyroid-related disease in the EMR were defined as controls. There were no abnormal values in the thyroid-related tests. We kept only one person from the same family in the control and case groups. We determined the members of the same family based on the results of Identical-by-state (IBS)/Identical-by-descent (IBD) (IBS/IBD > 0.25: is the coefficient used to calculate kinship, which we used to exclude people from the same family to ensure independence between samples) computation using PLINK 1.9 (–logistic, –covar sex and PC1~PC4). Finally, we randomly divided the subjects into two cohorts (70% for discovery cohort, 30% for replication cohort), divided the subjects into two groups (cases and controls) based on clinical annotation. There were 1,850 cases and 21,499 controls in the discovery cohort. There were 917 cases and 10,743 controls in the replication cohort. Logistic regression with multiple covariates was used to analyze the data. The covariates used in the logistic regression were sex and PC1–PC4. PC1 to PC4 were the results of principal component analysis (PCA) analysis using PLINK 1.9. We also adjusted for statistical significance. We plotted the Manhattan plot and quantile-quantile (QQ) plot with the *p-*value using R studio.

### Statistical analysis

Statistical analysis was performed according to our previous study (14). Comparisons between two groups was performed using the Student's *t*-test. Statistical comparisons of more than two groups were performed using one-way analysis of variance (ANOVA). In all cases, *p* < 0.05, was considered to be statistically significant.

## Result

**Figure 2:**
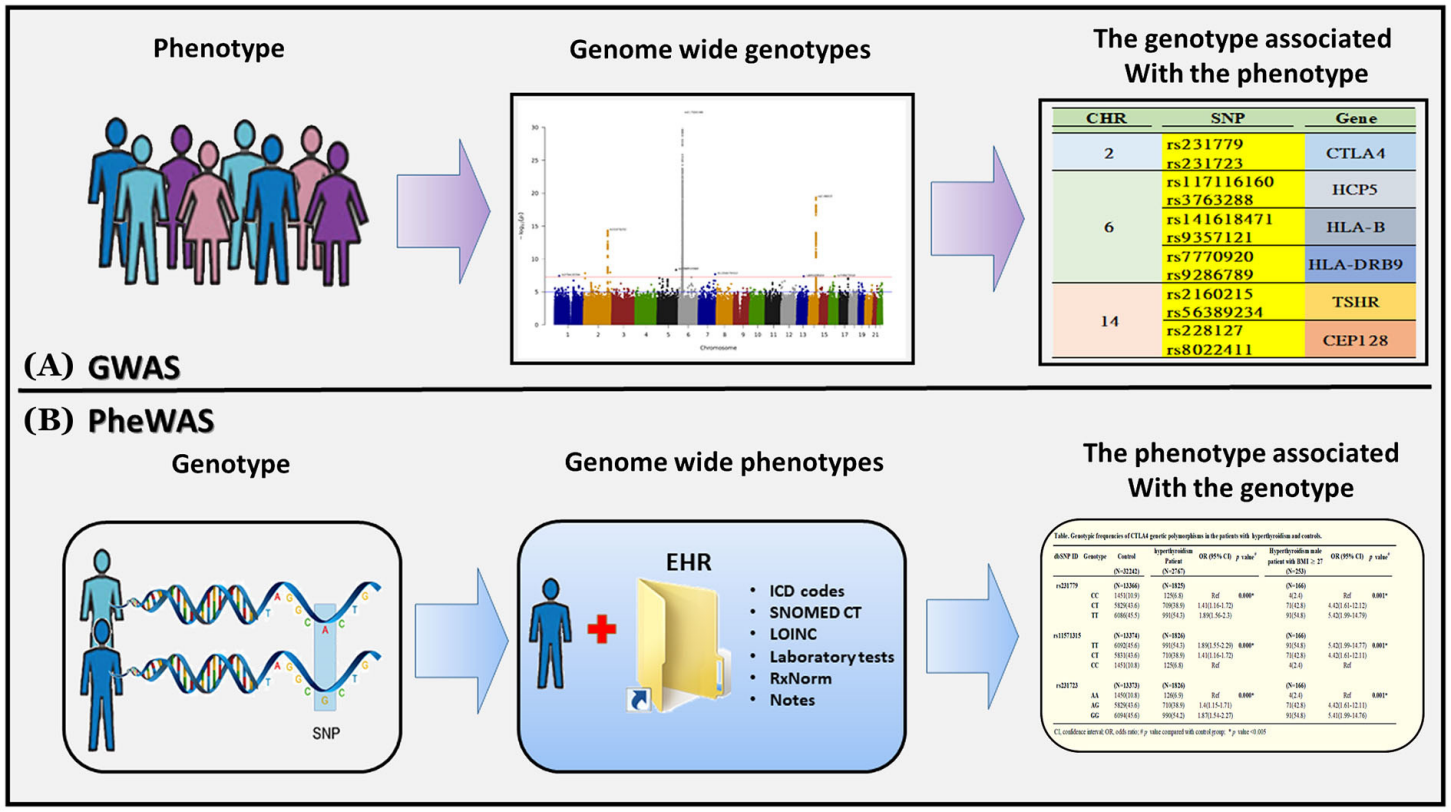
Genome-wide association studies (GWAS) and phenome-wide association studies (PheWAS). **(A)** A GWAS begins with a phenotype of interest and systematically analyzes variants across the entire genome (i.e., “genome-wide”) for association to the phenotype. GWAS can identify multiple genetic associations to a phenotype in complex or polygenic traits. **(B)** A PheWAS begins with a genetic variant of interest and systematically analyzes many phenotypes (i.e., “phenome-wide”) for association to the genotype. PheWAS has the ability to identify pleiotropy or multiple independent phenotypes associated with a single genetic variant.

We followed a flowchart for EMR data mining and the GWAS pipeline (Figure 1) and an Abstract Graph representation for research concept is shown in Figure 2. GWAS analyses with hyperthyroidism were performed on the discovery batch of 23,349 individuals included from 1992. The replication batch consisted of 11,660 individuals recruited from 1992 (Table 1). The same exclusion criteria were applied to both batches.

**Table 1:** Descriptive information on the discovery and replication batches.

**Sample characteristics**		
**Batch name**	**Discovery (*n* = 23,349)**	**Replication (*n* = 11,660)**
Sex (M/F) (%)	10,289 (44.1) / 13,060 (55.9)	5,199 (44.6)/6,461 (55.4)
Age (SD)	50.0 (19.497)	49.87 (19.372)
BMI (SD)	25.95 (5.991)	25.91 (5.905)
Hyperthyroidism	1,850 (7.9)	917 (7.9)

Genome-wide association studies (GWAS) analyses with hyperthyroidism were performed on a discovery batch comprising 23,349 individuals included from 1992 to 2019. The replication batch consisted of 11,660 individuals recruited from 1992 to 2019. The same exclusion criteria were applied to both batches.

**Figure 3:**
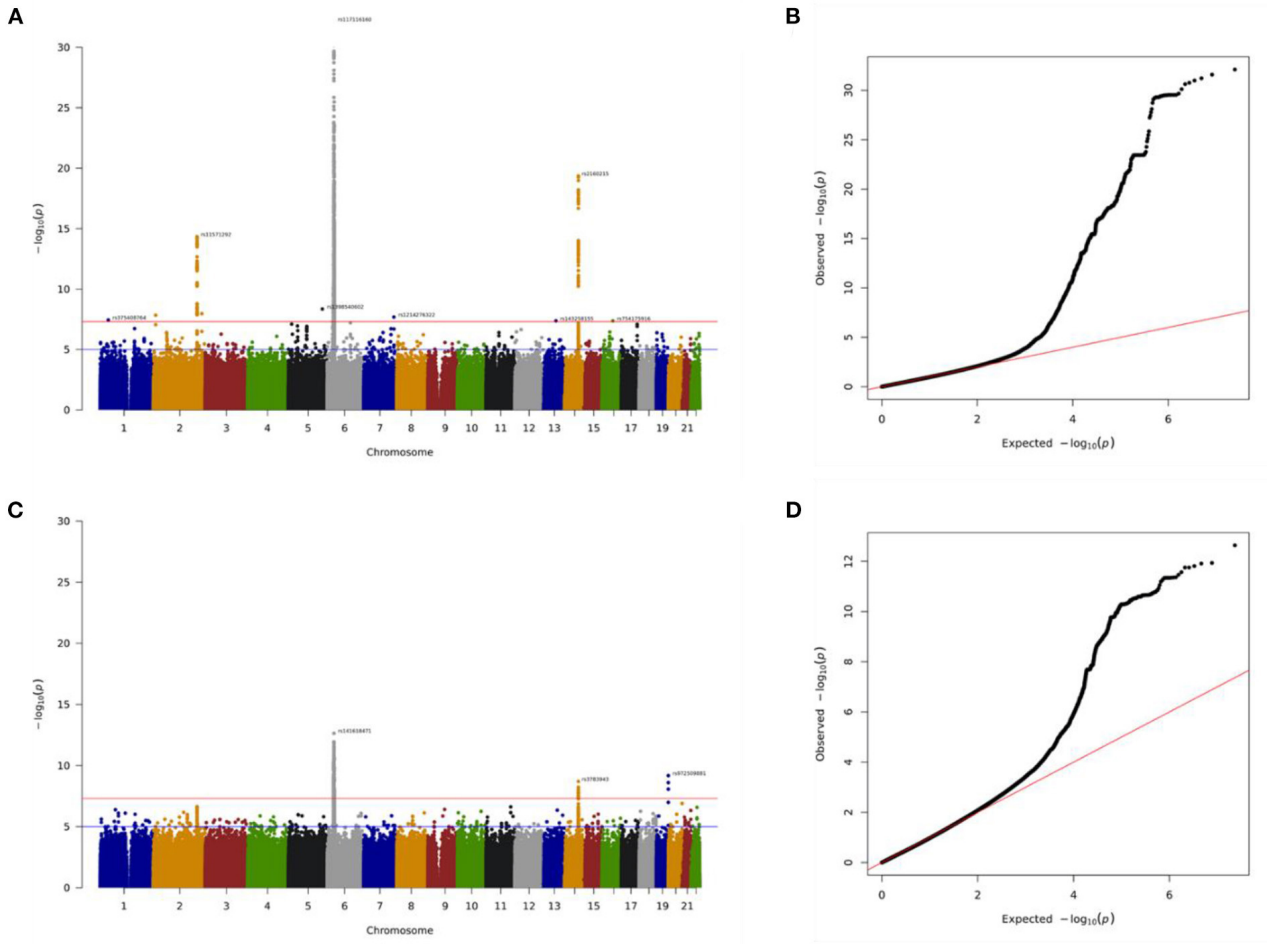
Association of genome-wide variants with hyperthyroidism diagnosed in discovery batch **(A,B)** and replication **(C,D)** batch using Manhattan plot **(A,C)** and QQ plot **(B,D)** analysis. In Manhattan plot analysis, single nucleotide polymorphism (SNP) that passed quality control are plotted on the X-axis according to their chromosomal positions against Y-axis (- log_10_ *p*-value). The upper and lower dotted lines indicate the genome-wide significance threshold (*p* = 5.0×10^−8^) and the cut-off level for selecting SNPs for replication study (*p* = 1.75×10^−5^), respectively.

The total genotyping rate of the remaining samples was 0.992366. A total of 3,034,044 variants and 35,009 people passed the filters and quality control among the remaining phenotypes. There were 23,349 people, including 1,850 cases and 21,499 controls in the discovery cohort, and 11,660 people, including 917 cases and 10,743 controls in the replication cohort. Manhattan plot is used to visualize GWAS analysis. The genome-wide significance level was set at *p* = 5 × 10^−8^ in the discovery batch (upper red line, Figure 3A) and *p* = 1.75 × 10^−5^ in the replication batch (upper red line, Figure 3C). The association of these single nucleotide polymorphisms (SNPs) that passed quality control are plotted on the X-axis according to their chromosomal positions against Y-axis (- log_10_ *p*-value). We also used QQ plots for genome-wide association analysis to investigate the correlation between hyperthyroidism patients and controls in the discovery and replication cohorts (Figures 3B,D). As new loci within the hyperthyroidism patients were identified by GWAS, we proved the associations with allelic variants of these new loci in linkage disequilibrium were shown to be stronger than previously observed associations (Supplemental material 1, SP1).

In this present study, we identified more than 1,500 SNPs associate with hyperthyroidism (SP2). For reducing the numbers and focused on the most significantly top 10 genes. Our GWAS data identified 44 novel genomic risk markers in 10 loci on chromosomes 2, 6, and 14 with the threshold of *p* < 5 × 10^−14^ in discovery analysis and *p* < 1.75 × 10^−5^ in replication analysis, including genes CTLA4, HCP5, HLA-B, POU5F1, CCHCR1, HLA-DRA, HLA-DRB9, TSHR, RPL17P3, and CEP128. The genes that showed a significant difference in our study have all been considered in the involvement of disease in previous studies (Table 2).

**Table 2:** Lead SNPs from the discovery- and replication-analysis.

**CHR**	**SNP**	**A1/A2**	***p*** **-Value (GWAS results)**	***p*** **-Value (Replication)**	**Gene**	**Function of gene**	**Involvement in disease**
2	rs1427680	G/A	2.07E-14	8.15E-06	CTLA4	Inhibitory receptor acting as a major negative regulator of T-cell responses (15, 16).	Systemic lupus erythematosus (SLE) (17)
	rs736611	C/T	2.19E-14	9.96E-06			Diabetes mellitus, insulin-dependent, 12 (IDDM12) (18)
	rs11571315	T/C	2.19E-14	9.43E-06			Celiac disease 3 (CELIAC3) (19)
	rs231723	A/G	3.24E-14	9.25E-06			Autoimmune lymphoproliferative syndrome 5 (ALPS5) (20)
6	rs117116160	C/T	7.73E-33	1.23E-12	HCP5	HCP5 (HLA Complex P5) is an RNA Gene, and is affiliated with the lncRNA class.	Acquired immunodeficiency syndrome (21)
	rs117884751	T/A	2.56E-32	1.76E-12			Thyroid gland follicular carcinoma (22)
	rs3763287	C/A	5.25E-25	1.41E-11			
	rs114202986	T/A	4.60E-24	3.45E-10			
	rs3763288	G/A	5.04E-24	3.88E-10			
	rs141618471	A/G	1.01E-31	2.31E-13	HLA-B	HLA-B (Major Histocompatibility Complex, Class I, B) is a Protein Coding gene.	Stevens-Johnson syndrome (SJS) (23)
	rs9378228	G/T	1.70E-31	4.48E-12			Spondyloarthropathy 1 (SPDA1) (24)
	rs12524692	T/A	7.74E-29	1.76E-12			
	rs72860306	C/T	5.84E-28	2.16E-11			
	rs9357121	T/G	9.24E-22	4.98E-12			
	rs117588763	C/T	2.88E-30	3.55E-11	POU5F1	Critical for early embryogenesis and for embryonic stem cell pluripotency (25).	Embryonal carcinoma (26)
	rs9357112	A/G	2.88E-30	3.55E-11			Teratoma (27)
	rs9357114	T/G	2.88E-30	3.55E-11			
	rs9348855	A/C	2.88E-30	3.55E-11			
	rs4713439	A/G	3.47E-30	4.10E-11			
	rs28652698	G/A	3.57E-28	2.06E-11	CCHCR1	Critical for early embryogenesis and for embryonic stem cell pluripotency.	Psoriasis (28)
	rs28383832	(-/CGCC)	1.70E-20	6.47E-10			Psoriasis 1 (PSORS1) (29)
	rs1265082	G/A	1.84E-20	4.21E-10			
	rs1265113	C/G	1.84E-20	4.21E-10			
	rs9469112	C/T	3.30E-26	1.71E-10	HLA-DRA	HLA-DRA (Major Histocompatibility Complex, Class II, DR Alpha) is a Protein Coding gene.	Graham-little-piccardi-lassueur syndrome (30)
	rs16822660	T/C	1.36E-22	1.94E-08			Penicillin allergy (31)
	rs9469113	G/A	2.50E-21	1.55E-07			
	rs7770920	T/A	3.51E-24	1.70E-10	HLA-DRB9	HLA-DRB9 [Major Histocompatibility Complex, Class II, DR Beta 9 (Pseudogene)] is a Pseudogene.	Rheumatoid arthritis (32)
	rs6457596	C/T	3.51E-24	1.70E-10			Vogt-koyanagi-harada disease (33)
	rs111573974	(-/G)	3.51E-24	1.70E-10			Multiple sclerosis (34)
	rs6924760	A/G	3.52E-24	1.71E-10			
	rs9286789	T/G	3.52E-24	1.71E-10			
14	rs2160215	T/A	4.45E-20	8.99E-09	TSHR	Plays a central role in controlling thyroid cell metabolism (By similarity) (35).	Hypothyroidism, congenital, non-goitrous, 1 (CHNG1) (36)
	rs1023586	T/C	4.45E-20	8.99E-09			Familial gestational hyperthyroidism (HTFG) (37)
	rs28414437	A/C	1.05E-19	1.25E-08			Hyperthyroidism, non-autoimmune (HTNA) (38)
	rs11159479	C/T	1.68E-18	2.98E-08			
	rs56389234	G/A	1.68E-18	2.98E-08			
	rs4903962	A/G	6.08E-19	1.63E-08	RPL17P3	RPL17P3 (Ribosomal Protein L17 Pseudogene 3) is a Pseudogene.	Thyroid (39)
	rs2268459	A/G	6.61E-19	4.76E-08			
	rs12323356	A/C	1.05E-18	1.72E-08			
	rs228127	G/A	9.84E-15	3.16E-06	CEP128	CEP128 (Centrosomal Protein 128) is a Protein Coding gene.	Hypothyroidism, Congenital, Non-goitrous, 1 (CHNG1) (40)
	rs7154132	C/T	1.05E-14	6.05E-06			Hyperthyroidism, Non-autoimmune (HTNA) (41)
	rs35176982	(-/AA)	1.40E-14	4.79E-06			
	rs1025253	G/A	1.46E-14	4.25E-06			
	rs8022411	G/T	1.46E-14	4.25E-06			

Briefly, ***CTLA4*:** Function of gene: Inhibitory receptor acting as a major negative regulator of T-cell responses (15, 16). Involvement in disease: Systemic lupus erythematosus (SLE) (17), diabetes mellitus, insulin-dependency, 12 (IDDM12) (18), celiac disease 3 (CELIAC3) (19), autoimmune lymphoproliferative syndrome 5 (ALPS5) (20). ***HCP5*:** Function of gene: *HCP5* (HLA Complex P5) is an RNA gene and is affiliated with the lncRNA class. Involvement in disease: acquired immunodeficiency syndrome (21), thyroid glandular carcinoma (22). ***HLA-B*:** Function of gene: *HLA-B* (major histocompatibility complex, class I, B) is a protein-coding gene. Involvement in disease: Stevens-Johnson syndrome (SJS) (23) and Spondyloarthropathy 1 (SPDA1) (24). ***POU5F1*:** Function of gene: Critical for early embryogenesis and embryonic stem cell pluripotency (25). Involvement in disease: Embryonal carcinoma (26), Teratoma (27). ***CCHCR1*:** Function of gene: Critical for early embryogenesis and embryonic stem cell pluripotency. Involvement in disease: Psoriasis (28), Psoriasis 1 (PSORS1) (29). ***HLA-DRA*:** Function of gene: HLA-DRA (major histocompatibility complex, class II, DR alpha) is a protein-coding gene. Involvement in disease: Graham-Little-Piccardi-Lassueur Syndrome (30), Penicillin Allergy (31). ***HLA-DRB9*:** Function of gene: *HLA-DRB9* [major histocompatibility complex, class II, DR beta 9 (pseudogene)] is a pseudogene. Involvement in disease: rheumatoid arthritis (32), Vogt-Koyanagi-Harada disease (33), multiple sclerosis (34). ***TSHR*:** Function of gene: plays a central role in controlling thyroid cell metabolism (by similarity) (35). Involvement in disease: Hypothyroidism, congenital, non-goitrous, 1 (CHNG1) (36), familial gestational hyperthyroidism (HTFG) (37), hyperthyroidism, non-autoimmune (HTNA) (38). ***RPL17P3*:** Function of gene: *RPL17P3* (ribosomal protein L17 pseudogene 3) is a pseudogene. Involvement in disease: thyroid (39). ***CEP128*:** Function of gene: *CEP128* (Centrosomal Protein 128) is a protein-coding gene. Involvement in disease: Hypothyroidism, Congenital, Nongoitrous, 1 (CHNG1) (40), Hyperthyroidism, Non-autoimmune (HTNA) (41).

Based on the prevalence of comorbidities among our study population, we further conducted a comorbidity analysis of our results using EMR data. A total of 2,767 subjects with a hyperthyroidism diagnosis (International Classification of Diseases, 9th Revision, Clinical Modification [ICD-9-CM] 242.90, 242.00, 242.900 or ICD10-code E05.0), with at least one TSH and free T4 or total T4 value, and with genotyping information were identified as subjects with hyperthyroidism (the case group). The gender were grouped by the available data in the study. Obesity in this study was defined as body mass index (BMI) ≥ 27 kg/m^2^, according to the Ministry of Health and Welfare of Taiwan. As shown as Supplementary material 3 (SP3), the extracted comorbidities were defined by the studies with disease diagnosis (ICD code). We observed that the incidence of thyroid storm in hyperthyroidism individuals was 1.3% (36/2767). We also observed that the risk of stroke in male individuals with hyperthyroidism was significantly higher than that female individuals with hyperthyroidism (*p* < 0.05, Table 3). Similar results were observed for stroke, heart disease, diabetes, and hypertension with statistical significance (*p* < 0.05, Table 4). Compared with normal body weight, individuals with a body mass index (BMI) of >28 (609/2556, 23.83%) also increased the risk of stroke, heart disease, diabetes, and hypertension in patients with hyperthyroidism, and the data were statistically significant (*p* < 0.05, Table 5). Moreover, a higher incidence of stroke (4/36, 11.1%) was observed in hyperthyroidism individuals with thyroid storm. Our data yield a strong correlation between hyperthyroidism patients with thyroid storm and stroke; the data were statistically significant (*p* < 0.05, Table 4).

**Table 3:** Comorbidity analysis in patients with hyperthyroidism using electronic medical record (EMR) data by gender.

	**Patients with hyperthyroidism** **(*****n*** = **2,767)**		***p-*** **Value**
	**Male (*****n*** = **637)**	**Female (*****n*** = **2, 130)**	
Thyroid storm	5 (0.8)	31 (1.5)	0.234
Cancer	22 (3.5)	78 (3.7)	0.904
Heart disease	33 (5.2)	58 (2.7)	0.003^a^
Osteoporosis	0 (0)	8 (0.4)	0.211
Infertility	3 (0.5)	26 (1.2)	0.122
Stroke	32 (5.0)	61 (2.9)	0.012^a^
Diabetes	93 (14.6)	168 (7.9)	0.000^a^
Hypertension	49 (7.7)	99 (4.6)	0.005^a^
Hyperlipidemia	10(1.6)	27(1.3)	0.557
gallstone	12(1.9)	26(1.2)	0.242

a Significant difference at p < 0.05.

**Table 4:** Comorbidity analysis in hyperthyroidism patients with thyroid storm using electronic medical record (EMR) data.

	**Hyperthyroidism patient with thyroid storm** **(*****n*** = **36)**	**Hyperthyroidism patient without thyroid storm** **(*****n*** = **2,731)**	***p*** **-Value**
Cancer	2 (5.6)	98 (3.6)	0.376
Heart disease	1 (2.8)	90 (3.3)	1.000
Osteoporosis	1 (2.8)	7 (0.3)	0.100
Infertility	1 (2.8)	28 (1.0)	0.317
Stroke	4 (11.1)	89 (3.3)	0.031^a^
Diabetes	3 (8.3)	258 (9.4)	1.000
Hypertension	3 (8.3)	145 (5.3)	0.439
Hyperlipidemia	0 (0)	37 (1.4)	1.000
Gallstone	1 (2.8)	37 (1.4)	0.394

a Significant difference at p <0.05.

**Table 5:** Comorbidity analysis in patients with hyperthyroidism using electronic health record (EHR) data of BMI.

	**Patient with hyperthyroidism** **(*****n*** = **2,556)**^b^		***p*** **-Value**
	**BMI** ≧**27** **(*****n*** = **773)**	**BMI**<**27** **(*****n*** = **1,783)**	
Thyroid storm	11 (1.4)	24 (1.3)	0.8546
Cancer	38 (4.9)	57 (3.2)	0.0402
Heart disease	45 (5.8)	40 (2.2)	0.0000^a^
Osteoporosis	3 (0.4)	5 (0.3)	0.7046
Infertility	9 (1.2)	20 (1.1)	1.0000
Stroke	39 (5.0)	51 (2.9)	0.0072^a^
Diabetes	127 (16.4)	116 (6.5)	0.0000^a^
Hypertension	81 (10.5)	54 (3.0)	0.0000^a^
Hyperlipidemia	14 (1.8)	21 (1.2)	0.2006
Gallstone	19 (2.5)	16 (0.9)	0.0028^a^

a With significant differences and P < 0.05.

b 211 Patients without HER data of BMI.

## Discussion

In the present study, we identified 44 novel variants in 10 loci associated with hyperthyroidism, including *CTLA4, HCP5, HLA-B, POU5F1, CCHCR1, HLA-DRA, HLA-DRB9, TSHR, RPL17P3*, and *CEP128*. To consider differences in racial backgrounds, and proved that these SNPs is really significant associate with the disease. We further compared the Taiwan Biobank data. Such as Supplementary material 4 (SP4), the SNPs data from Taiwan Biobank indicated that these candidate SNPs in our study were indeed significant difference from those in Taiwan without hyperthyroidism. First, Principal components (PC1-10) were added into GWAS to exclude the effect of racial backgrounds. Second, the subjects enrolled from Taiwan Biobank are Han Chinese in Taiwan and used as general controls. Therefore, the significantly difference of genotype distributions for those selected SNPs between Taiwan Biobank population (as general control) and subjects with hyperthyroidism in our study population could provide evidences that these SNPs are associated with hypertension. To our knowledge, five novel genes that have never before been discussed were found to be associated with hyperthyroidism: *POU5F1, CCHCR1, HLA-DRB9, RPL17P3*, and *CEP128*. Here, we show the biological function and involvement of disease in these genes, which have been discussed previously (Table 2). The related pathways of these candidate genes were then analyzed by DAVID with the recently updated Kyoto Encyclopedia of Genes and Genomes (KEGG) database (https://www.genome.jp/kegg/pathway.html). Our data showed that these genes were significantly associated in pathways related to autoimmune thyroid disease (SP5).

Comorbidity analysis in patients with hyperthyroidism and thyroid storm is shown in Table 4. There were no differences in cancer, heart disease, osteoporosis, infertility, diabetes, hypertension, hyperlipidemia, or gallstones. We observed that the percentage of stroke in hyperthyroidism patients with thyroid storm was much higher than that in hyperthyroidism patients without thyroid storm (*p* < 0.05). Briefly, our data indicate that hyperthyroidism patients with thyroid storm may have a higher risk of developing stroke (Table 4).

Hormones and the cardiovascular system are strongly associated, and disorders of hormonal secretion may lead to increased cardiovascular risk (42, 43). In addition to these well-known effects, there is increasing evidence that hyperthyroidism may accelerate atherosclerosis (44, 45). Endothelial dysfunction, hypercoagulability, and thyroid autoimmunity have been suggested as potential contributors (45–48). Thyroid hormones exert important effects on the cardiovascular system, as demonstrated by the adverse clinical effects that can occur in states of hyperthyroidism and hypothyroidism. Thyroid disorders can impair cardiovascular risk factors, such as those included in the definition of metabolic syndrome (49, 50). Indeed, excess as well as lack of thyroid hormone has been linked to alterations in cardiovascular hemodynamics (51), modifications of heart rhythm (52, 53), and arterial wall structure (54–56). While the effects of thyroid hormone excess on cardiovascular risk factors are clear for some of them, others are still debatable (57, 58). In the United States, stroke is the third leading cause of death, and ~795,000 people suffer from a new or recurrent stroke annually (59). The prevalence of stroke in Taiwan is reported to be 14.27–19.3 per 1,000 person-years; stroke is the most common cause of complex disability in Taiwan (60, 61). In a Taiwan National Health Insurance Research Database (NHIRD) study, Sheu et al. reported an increased risk of ischemic stroke in young patients with hyperthyroidism (1%) compared with a comparable population without thyroid disease (0.7%) after adjusting for AF (62). In this present study, we defined the significant common genetic risk factors in patients with hyperthyroidism. It might be contributed to the disease early diagnosis with precision medicine.

There was a significant difference in HLA gene loci in our results (Table 2). The human leukocyte antigen (HLA) system, located in the major histocompatibility complex (MHC) on chromosome 6, is highly polymorphic. This region has been shown to be important in human diseases, adverse drug reactions, and organ transplantation. For instance, HLA-B^*^46:01 is associated with Graves' disease in Taiwan (63). However, the HLA subtype cannot be predicted using a single-nucleotide polymorphism (SNP)-based tagging approach. To understand the relationship between HLA subtypes and diseases, machine learning methods such as HIBAG can be used to better predict HLA subtypes (64). The detailed relationship between these HLA subtypes should be studied in the future.

In order to connect the data between GWAS and PheWAS and further demonstrate the SNPs we identified could predict the risk of comorbidities associated with hyperthyroidism. We analysis the genotypic frequencies of CTLA4 genetic polymorphisms in hyperthyroidism patients and controls (Table 6). Compared with controls, the statistically significant difference was observed in the genotype frequency distribution of CTLA4 rs231779, rs1427680, rs736611, rs11571315, and rs231723 SNPs. We observed the Odds ratio (OR) were from 1.40 to 1.90. We further included the data of gender and BMI ≧ 27 for analysis, the similar results was observed with statistically significant difference in the genotype frequency distribution of CTLA4 SNPs. However, there was a surprising finding in the section of Odds ratio (OR). Our data showed that the Odds ratio all increased in these five SNPs and the OR value was observed from 4.40 to 5.42 (Table 6).

**Table 6:** Genotypic frequencies of CTLA4 genetic polymorphisms in the patients with hyperthyroidism and controls.

**dbSNP ID**	**Genotype**	**Control** **(*****N*** = **32,242)**	**hyperthyroidism Patient** **(*****N*** = **2,767)**	**OR (95% CI)**	***p-*** **Value** ^#^	**Hyperthyroidism male patient with BMI** ≧**27** **(*****N*** = **253)**	**OR (95% CI)**	***p-*** **Value** ^#^
rs1427680		(*N* = 13,357)	(*N* = 1,822)			(*N* = 165)		
	GG	6,083 (45.5)	989 (54.3)	1.90 (1.56–2.3)	0.000*	90 (54.6)	5.35 (1.96–14.59)	0.001*
	GA	5,828 (43.6)	709 (38.9)	1.42 (1.16–1.73)		71 (43.0)	4.40 (1.61–12.08)	
	AA	1,446 (10.9)	124 (6.8)	Ref		4 (2.4)	Ref	
rs736611		(*N* = 13,360)	(*N* = 1,824)			(*N* = 165)		
	CC	6,086 (45.6)	989 (54.2)	1.90 (1.56–2.30)	0.000*	90 (54.6)	5.35 (1.96–14.58)	0.001*
	CT	5,828 (43.6)	711 (39.0)	1.42 (1.17–1.74)		71(43.0)	4.40 (1.61–12.08)	
	TT	1,446 (10.8)	124 (6.8)	Ref		4 (2.4)	Ref	
rs11571315		(*N* = 13,374)	(*N* = 1,826)			(*N* = 166)		
	TT	6,092 (45.6)	991 (54.3)	1.89 (1.55–2.29)	0.000*	91 (54.8)	5.42 (1.99–14.77)	0.001*
	CT	5,831 (43.6)	710 (38.9)	1.41 (1.16–1.72)		71 (42.8)	4.42 (1.61–12.11)	
	CC	1,451 (10.8)	125 (6.8)	Ref		4 (2.4)	Ref	
rs231723		(*N* = 13,373)	(*N* = 1,826)			(*N* = 166)		
	AA	1,450 (10.8)	126 (6.9)	Ref	0.000*	4 (2.4)	Ref	0.001*
	AG	5,829 (43.6)	710 (38.9)	1.40 (1.15–1.71)		71 (42.8)	4.42 (1.61–12.11)	
	GG	6,094 (45.6)	990 (54.2)	1.87 (1.54–2.27)		91 (54.8)	5.41 (1.99–14.76)	

CI, confidence interval; OR, odds ratio; # p-value compared with control group;

* p < 0.005.

In conclusion, our findings strongly suggest an association between 44 genetic variants in ten loci and hyperthyroidism susceptibility, and that these genes contribute to the genetic background of hyperthyroidism pathogenesis. Moreover, our data indicate that hyperthyroidism patients with thyroid storm may have a higher risk of developing stroke. We also connected the data between GWAS and PheWAS and demonstrated the SNPs we identified could predict the risk of comorbidities associated with hyperthyroidism. These findings should prompt specific considerations for the diagnosis and treatment of patients with hyperthyroidism, especially in preventing stroke.

## Data availability statement

The original contributions presented in the study are publicly available. This data can be found at: https://my.locuszoom.org/gwas/239175/?token=ba633ab327054c3f82c34f7d3cf346d5; https://my.locuszoom.org/gwas/292022/?token=51201ae60be34ae6a8859fe84e908c0c.

## Ethics statement

All clinical information was collected from the electronic medical records (EMRs) of CMUH and approved by the respective Ethical Committees of CMUH (CMUH110-REC1-095). The patients/participants provided their written informed consent to participate in this study.

## Author contributions

S-YC had full access to all of the data in the study and takes responsibility for the integrity of the data and the accuracy of the data analysis. T-YL, W-LL, T-YW, S-YC, and F-JT: concept and design. C-JC, J-GC, Y-CC, H-FL, H-HY, and S-YC: acquisition, analysis, or interpretation of data. T-YL, W-LL, T-YW, S-YC, and F-JT: drafting of the manuscript. C-JC: statistical analysis. All authors: critical revision of the manuscript for important intellectual content, read, and approved the final manuscript.

## Funding

This work was supported by China Medical University Hospital (DMR-108-138 and DMR-109-141).

## Conflict of interest

The authors declare that the research was conducted in the absence of any commercial or financial relationships that could be construed as a potential conflict of interest.

## Publisher's note

All claims expressed in this article are solely those of the authors and do not necessarily represent those of their affiliated organizations, or those of the publisher, the editors and the reviewers. Any product that may be evaluated in this article, or claim that may be made by its manufacturer, is not guaranteed or endorsed by the publisher.
