# Supplementary material for: Genome-wide association study of hyperthyroidism based on electronic medical record from Taiwan
Source: Front Med (Lausanne). 2022 Jul 27;9:830621. doi: 10.3389/fmed.2022.830621 (PMC9390483; doi:10.3389/fmed.2022.830621)
Supplement: Supplementary file 1 [file Presentation_1.PPTX]

## Slide 1
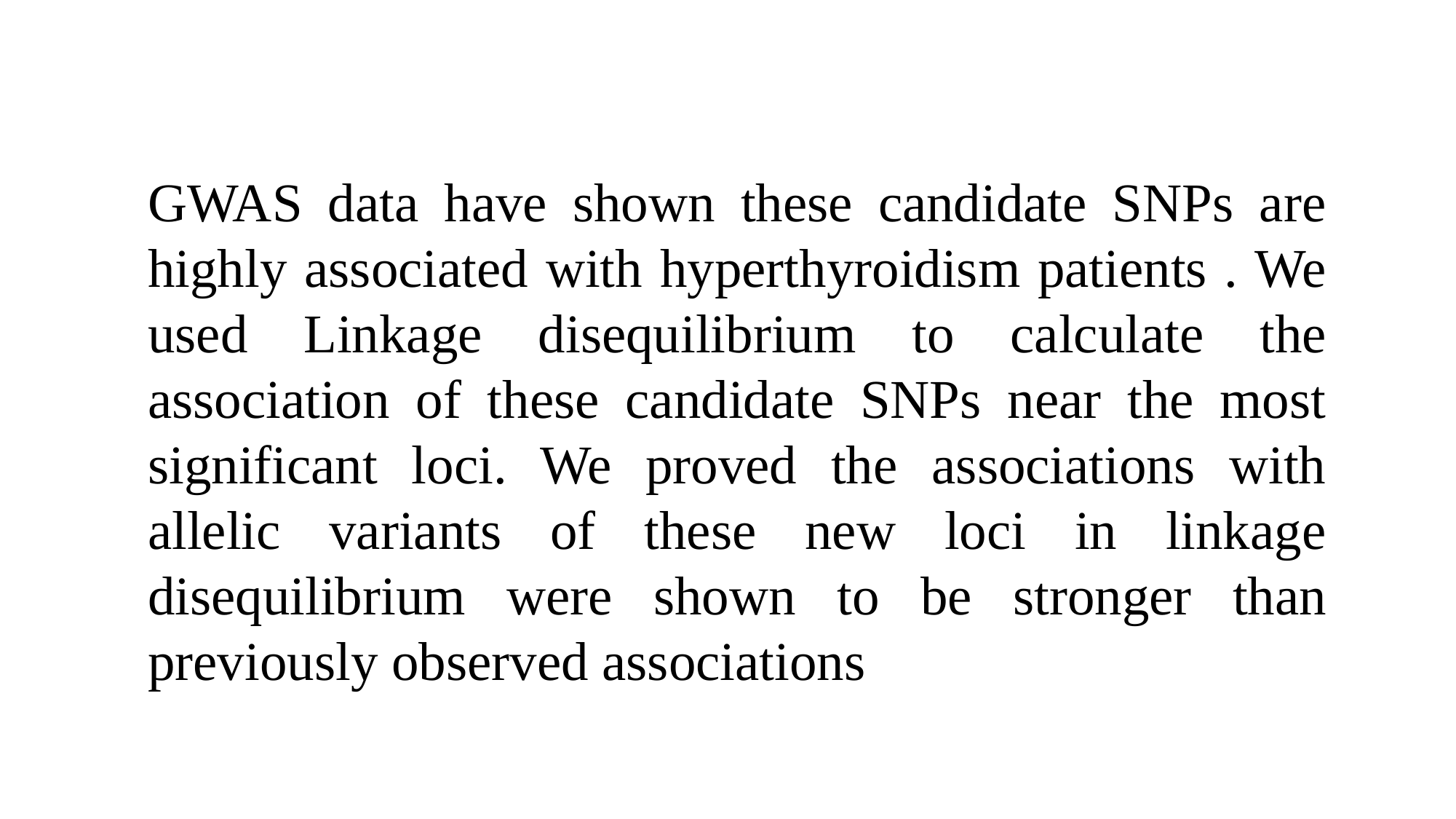

GWAS data have shown these candidate SNPs are highly associated with hyperthyroidism patients . We used Linkage disequilibrium to calculate the association of these candidate SNPs near the most significant loci. We proved the associations with allelic variants of these new loci in linkage disequilibrium were shown to be stronger than previously observed associations

## Slide 2
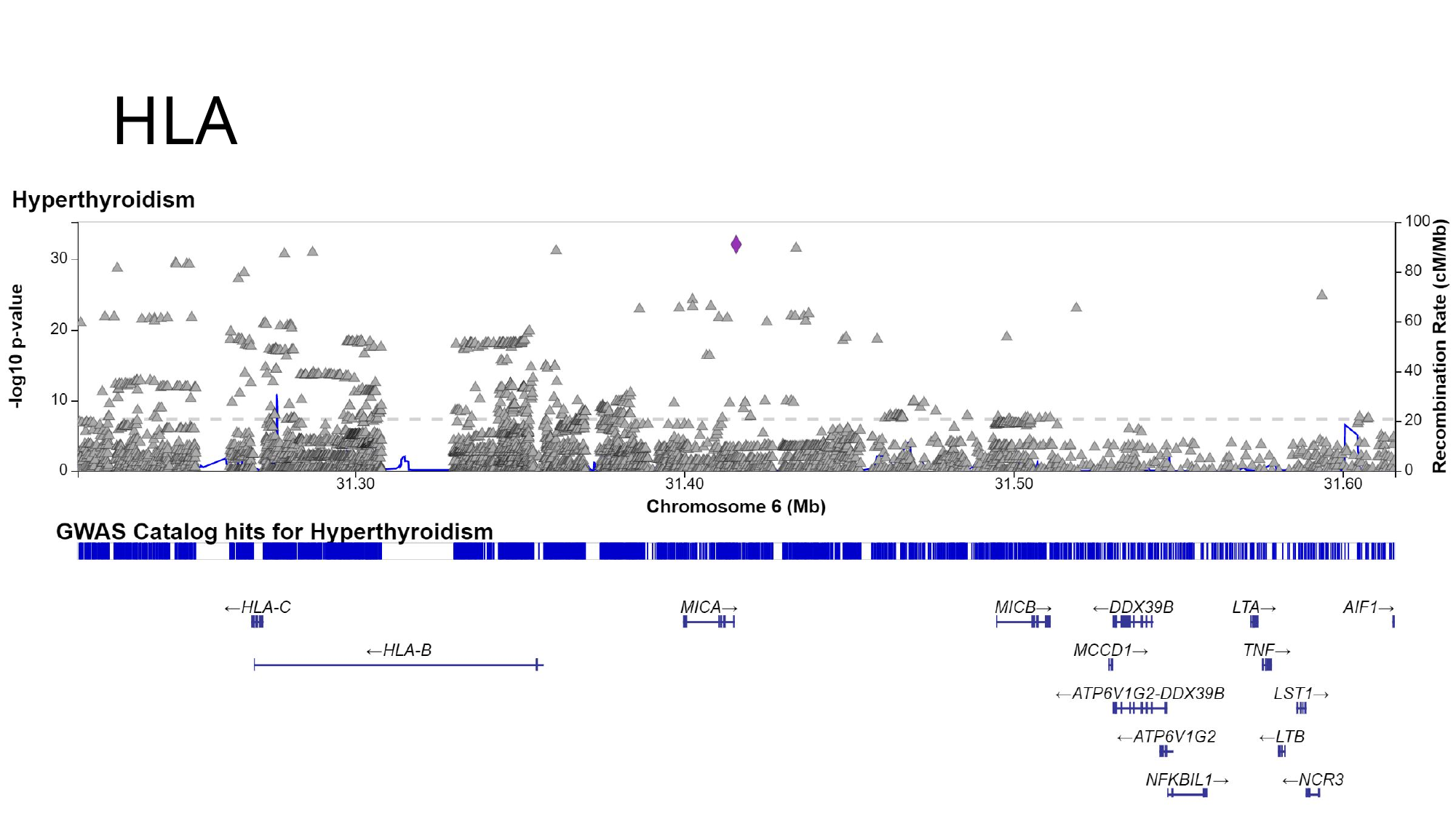

# HLA

## Slide 3
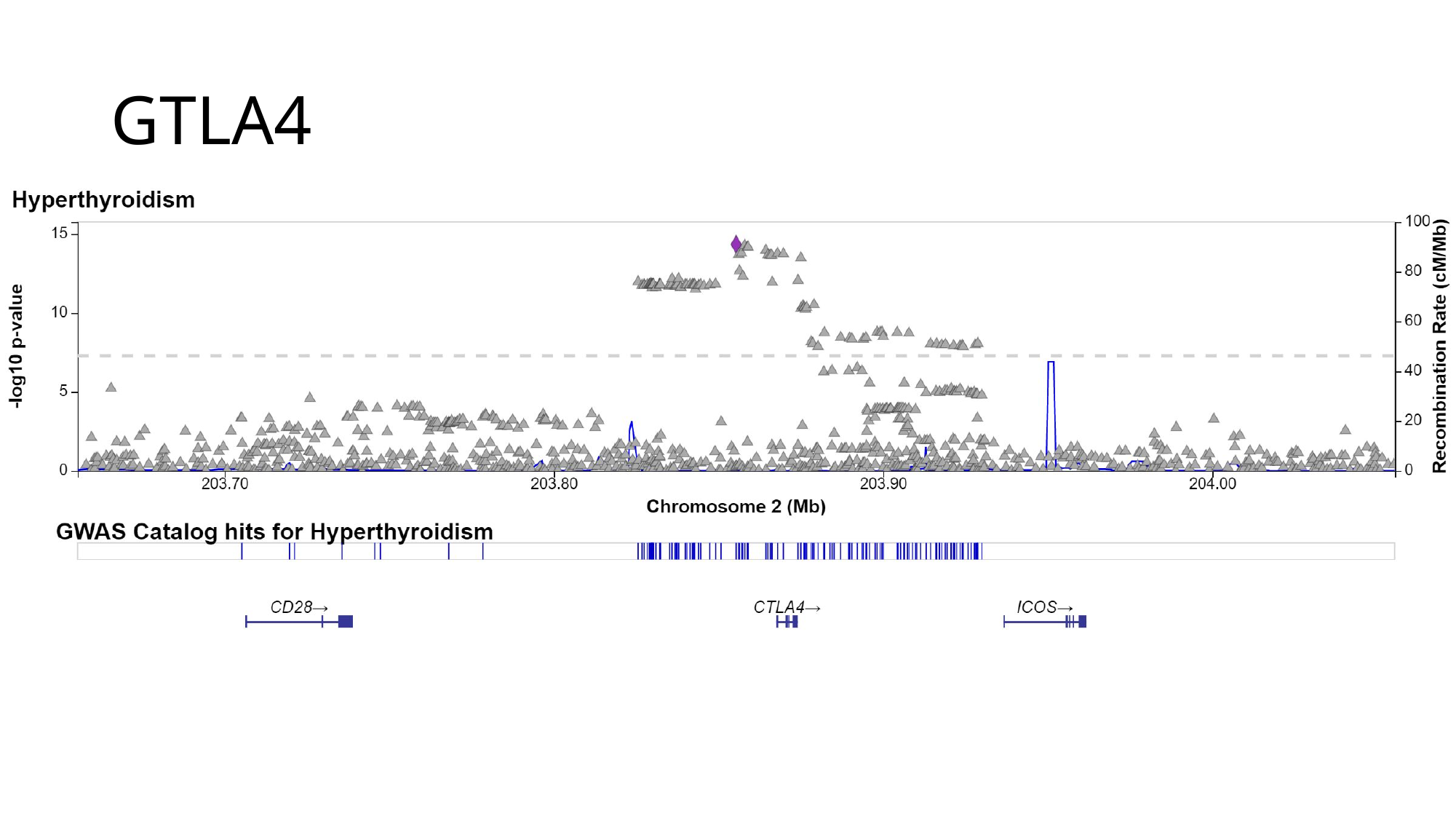

# GTLA4

## Slide 4
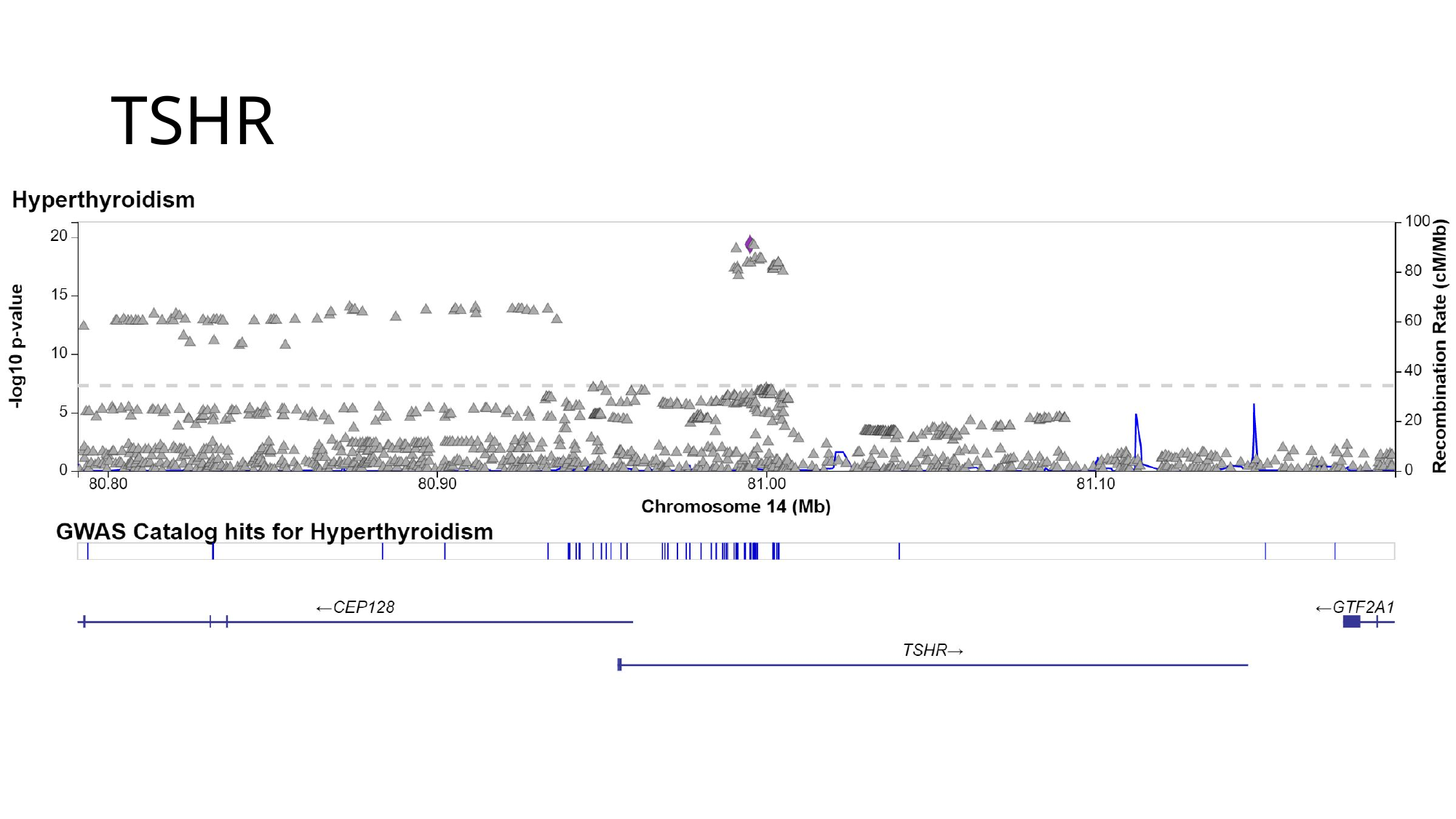

# TSHR
